# Supplementary material for: Assessment of COVID-19 data reporting in 100+ websites and apps in India
Source: PLOS Glob Public Health. 2022 Apr 15;2(4):e0000329. doi: 10.1371/journal.pgph.0000329 (PMC10022220; doi:10.1371/journal.pgph.0000329)
Supplement: S1 Text — (PDF) [file pgph.0000329.s001.pdf]

Assessment of COVID-19 data reporting in 100+ websites and apps in India

Varun Vasudevan[1¶], Abeynaya Gnanasekaran[1¶], Bhavik Bansal[2], Chandrakant Lahariya[3], Giridara Gopal Parameswaran[4], James Zou[5*]

[1]Institute for Computational & Mathematical Engineering, Stanford University, California, United States of America.

[2]All India Institute of Medical Sciences, New Delhi, India

[3]Public Policy and Health Systems Specialist, New Delhi, India

[4]Center for Disease Dynamics, Economics & Policy, New Delhi, India

[5]Department of Biomedical Data Science, Stanford University School of Medicine, California, United States of America

[*]Corresponding author

Email: jamesz@stanford.edu

[¶]These authors contributed equally to this work.

Supporting Information

A. Shortlisting digital platforms

If no relevant data was found on subnational government or health department websites and mobile applications, a google search was done using the phrases "<subnational> government covid website," "<subnational> government corona website," and "covid bed availability <subnational>." We parsed through the first two pages of search results and shortlisted all government sources. The government websites listed at help.covidtoday.in were also included.

The complete list of digital platforms that we shortlisted and checked are publicly available through the dataset released with this paper. At least two authors checked each platform independently and arrived at a consensus on what data is being reported. We excluded data reported on social media platforms due to their inherent limitations.[1]

B. Surveillance reporting score for each subnational

Table A: Surveillance reporting score for each subnational calculated using all the indicators proposed in [1]. Subnationals are sorted in the decreasing order of score.

Subnational	Surveillance reporting score
Nagaland	0.61
Kerala	0.57
Ladakh	0.52
Odisha	0.52
Punjab	0.52
Tamil Nadu	0.52
Puducherry	0.50
Tripura	0.50
West Bengal	0.50
Chhattisgarh	0.48
Gujarat	0.48
Haryana	0.48
Karnataka	0.48
Maharashtra	0.48

Uttarakhand	0.48
Andhra Pradesh	0.46
Jammu and Kashmir	0.46
Jharkhand	0.46
Meghalaya	0.46
Mizoram	0.46
Telangana	0.46
Madhya Pradesh	0.43
Rajasthan	0.43
Sikkim	0.43
Chandigarh	0.41
Andaman and Nicobar	0.39
Arunachal Pradesh	0.39
Assam	0.39
Delhi	0.39
Goa	0.39
Himachal Pradesh	0.39
Manipur	0.39
*Bihar	0.33
*Dadra and Nagar Haveli and Daman and Diu	0.33
*Lakshadweep	0.33
*Uttar Pradesh	0.33

*Data from these subnationals are available only through MyGov.

C. Calculating the amount of missing granular surveillance data

To get a handle on the scale of missing granular data, we narrow our focus on the reporting of age and gender for confirmed cases; and age, gender, and comorbidity for deaths. Among subnationals reporting these items, some disaggregate the cumulative numbers by the items; some disaggregate the daily numbers by the items, and the remaining report the items for each individual.

Considering all subnationals that report one or more of age, gender, and comorbidity, in any of the three forms mentioned above, we calculated the percentage of cases and deaths for which age, gender, and comorbidity distribution is available. As of 5 June 2020, India had over 28.6 million cases and over 344 thousand deaths. Among these, we have age and gender distribution for 13.75% and 15.24% of the cases, as shown in Fig 4A. Similarly, as of 5 June 2020, we have age, gender, and comorbidity distribution for 19.46%, 21.96%, and 28.65% of the deaths, respectively, as shown in Fig 4B. The calculations are as shown in the tables below.

Table B: Subnationals that report age and/or gender data for cases. Case numbers as of 5 June 2021 are taken from the MyGov app. Total cases in India = 28.6 million

Subnational	Total cases in subnational	Percentage of total cases in India = 100 x (Total cases in subnational / Total cases in India)
Nagaland	22387	0.078
Odisha	798699	2.793
Tamil Nadu	2.19E+06	7.657
Telangana	587664	2.055
Haryana	760914	2.661
Uttarakhand	332959	1.164

Table C: Calculation of percentage of cases for which age and gender distribution are available in India as of 5 June 2021.

Granular item	Subnationals reporting the granular item	Total %
Age	Nagaland (0.078) + Odisha (2.793) + Tamil Nadu (7.657) + Telangana (2.055) + Uttarakhand (1.164)	13.75
Gender	Nagaland (0.078) + Odisha (2.793) + Tamil Nadu (7.657) + Telangana (2.055) + Haryana (2.661)	15.24

Table D: Subnationals that report age, gender, and/or comorbidity data for deaths. Death numbers as of 5 June 2021 are taken from the MyGov app. Total deaths in India = 344082

Subnational	Total deaths in subnational	Percentage of total deaths in India = 100 x (Total deaths in subnational / Total deaths in India)
Karnataka	30895	8.979
Nagaland	416	0.121
Tamil Nadu	26128	7.594
Haryana	8605	2.501
Kerala	9510	2.764
Chhattisgarh	13162	3.825
Telangana	3346	0.972
West Bengal	16034	4.660

Table E: Calculation of percentage of deaths for which age, gender, and comorbidity distribution are available in India as of 5 June 2021.

Granular item	Subnationals reporting the granular item	Total %
Age	Karnataka (8.979) + Nagaland (0.121) + Tamil Nadu (7.594) + Kerala (2.764)	19.46
Gender	Karnataka (8.979) + Nagaland (0.121) + Tamil Nadu (7.594) + Haryana (2.501) + Kerala (2.764)	21.96
Comorbidity	Karnataka (8.979) + Nagaland (0.121) + Tamil Nadu (7.594) + Haryana (2.501) + Chhattisgarh (3.825) + Telangana (0.972) + West Bengal (4.660)	28.65

D. Additional notes on Figs 2 and 4

- Fig 2 shows a decrease in the *granular* surveillance reporting score for Tripura from assessment 1 in May 2020 (0.29) to assessment 2 in July 2020 (0.22). It is not because they **stopped** reporting a granular item. The reason is as follows. Tripura did not have any COVID-19 deaths until the completion of assessment 1. So, we excluded relevant granular indicators for deaths during assessment 1. However, by the time of assessment 2, Tripura had COVID-19 deaths, and their reporting did not have all the granular information. They got a lower score because we included all indicators in this assessment.

- Fig 4D: Chhattisgarh stopped reporting the comorbidity distribution for *total* deaths after the assessments in 2020. Instead, they are now reporting comorbidity distribution for *daily* deaths.
- Fig 4D: Karnataka stopped reporting the age distribution for *total* deaths after the assessments in 2020. However, they are continuing to report the age of *each* deceased person.

E. Suggestions on granular reporting of testing data

The number of cases reported by a subnational is a function of its testing capacity and testing coverage. We make a few suggestions to subnationals aimed at granular test reporting. Apart from reporting the number of tests and test positivity, subnationals could report the following. First, the reporting lag (interval between symptom onset and confirmation), which is crucial in estimating the effective reproductive number and understanding the demand in the testing capacity.[2] Second, test numbers and results disaggregated by symptomatic versus asymptomatic patients; contacts traced versus others; and type of testing (RT-PCR, rapid antigen, etc). This data is representative of the real-time community spread of the virus and how much the health system is in control.

F. Privacy violation in the reporting from Haryana

ORDER

Whereas it has been reported that following persons of District Jhajjar found positive for COVID-19 infection and admitted in Institutional isolation/home isolation as mentioned below.

S.No	Name of Patient (with age)	Address	Institutional Isolation or Home Isolation	Boundaries of Containment zone to be notified	Health facility centre to monitor the Patent	Houses and Population of Containment Zone	Remarks (New Containment and Extension)
1.	[redacted]	[redacted]	Home isolation	[redacted]	PHC Dujana	Houses: 08 Population : 30	Ext of order 392/MB Date 04.09.21
2.	[redacted]	[redacted]	Home isolation	[redacted]	PHC Nuna Majra	Houses: 15 Population :87	Ext of order 278/MB Date 04.07.21

Fig A: Screenshot of the document published by Haryana containing the name, age, gender, and address of COVID-19 cases. We blacked out personal information in the screenshot.

References for supporting information

1. Vasudevan V, Gnanasekaran A, Sankar V, Vasudevan SA, Zou J. Disparity in the quality of COVID-19 data reporting across India. BMC Public Health. 2021 Jun 24;21(1):1211.
2. Cori A, Kamvar Z, Stockwin J, Jombart T, Dahlqeist E, FitzJohn R, et al. EpiEstim v2.2-3: A tool to estimate time varying instantaneous reproduction number during epidemics [Internet]. 2021. Available from: https://github.com/mrc-ide/EpiEstim
