# Supplementary material for: Assessment of COVID-19 data reporting in 100+ websites and apps in India
Source: PLOS Glob Public Health. 2022 Apr 15;2(4):e0000329. doi: 10.1371/journal.pgph.0000329 (PMC10022220; doi:10.1371/journal.pgph.0000329)
Supplement: Attachment — Submitted filename: Response to Reviewers.docx [file pgph.0000329.s002.docx]

Response to Reviews

**Title**: Assessment of COVID-19 data reporting in 100+ websites and apps in India

**Authors**: Varun Vasudevan, Abeynaya Gnanasekaran, Bhavik Bansal, Chandrakant Lahariya, Giridara Gopal Parameswaran, James Zou

**Date:** Nov 23, 2021

# Journal Requirements

Please amend your detailed Financial Disclosure statement. This is published with the article, therefore should be completed in full sentences and contain the exact wording you wish to be published.

State what role the funders took in the study. If the funders had no role in your study, please state: “The funders had no role in study design, data collection and analysis, decision to publish, or preparation of the manuscript.”

Thank you. We have added the above sentence to our manuscript.

# Reviewer 1

## Overall Comment

The area is interesting but the manuscript does not follow the scientific guidelines of a research paper. It appears more like a book chapter. Authors need to cover the apps in detail and present a framework for new app-makers to follow through their research efforts. Currently only reporting is being done which does not add much value to the existing literature.

Thank you for the feedback. We want to clarify that the main goal and contribution of our paper is to quantify the quality of COVID-19 data reporting in India. This is an important and timely topic for public health. The app itself is only to visualize the quality scores and is not the main focus; and therefore we followed previous publications' visualization designs.[[1]](https://www.zotero.org/google-docs/?R6fAZi) Presenting a design framework for new app-makers is an interesting idea for future research.

Our paper systematically analyzes the COVID-19 data reporting from governments in India. Our analyses reveal a lack of granular data in the reporting of COVID-19 surveillance, vaccination, and vacant bed availability. As of 5 June 2021, age and gender distribution are available for less than 22% of cases and deaths, and comorbidity distribution is available for less than 30% of deaths. Amid rising concerns of undercounting cases and deaths in India, our results highlight a patchy reporting of granular data even among the reported cases and deaths. Furthermore, total vaccination stratified by healthcare workers, frontline workers, and age brackets is reported by only 14 out of India's 36 subnationals (states and union territories).

By identifying important weaknesses in current practice, we enable better reporting practices in the future. This can impact public health by improving trust, transparency and information flow. To the best of our knowledge, there is no study to systematically analyze surveillance, vaccination, and vacant bed availability reporting in India, and our work is the largest study of its kind. We present a comprehensive assessment of the quality of data reporting in 100+ national and subnational government digital platforms, both web and mobile. Through a semi-quantitative approach, we show the specifics and magnitude of missing granular data across India. It is extremely important to understand and document this because India experienced a severe second wave.

Moreover, we have developed a new framework in this work to assess the quality of vaccination reporting. While we have used the surveillance and vaccination reporting framework to study COVID-19 data reporting in India, the frameworks themselves are quite general and can be easily adapted to other diseases and countries. Therefore, we believe that our work adds a lot of value both in the short term and in the long term.

**Data availability:** The dataset that we curated for this study is made publicly available at <https://github.com/varun-vasudevan/CDRS-India/tree/master/study3_june_2021>.

**Statistical analysis:** We have added a five number summary of the surveillance reporting score and the granular surveillance reporting score: minimum, first quartile, median, third quartile, and maximum.

In writing this manuscript we have followed the formatting guidelines of the journal for Research Paper as provided in <https://journals.plos.org/globalpublichealth/s/submission-guidelines> for style, format and the organization of the manuscript. We have organized the manuscript into a beginning section (title, abstract, introduction), middle section (methods, results, discussion and conclusions) and ending section (acknowledgements, references and supporting information) as given in the guidelines.

# Reviewer 2

The authors present an important topic examining the quality of reporting of COVID-19 data (including surveillance, vaccination monitoring, and hospital bed availability) in over 100 government platforms (web and mobile) from India. The article flows well and there are limited typos. While the article shows great potential and the subject matter is important, there are several modifications that are suggested. In the current state, a revision and resubmission is recommended.

Thank you for your feedback. We have carefully updated the paper based on your suggestions.

## Introduction

Additional information about why this data is important and the potential impact it has for different stakeholder groups would strengthen the Introduction.

We have strengthened the introduction by adding text on how granular data is useful to the public and to researchers. See lines 56-68 on Page 3.

Newly added text:

Reporting granular COVID-19 data is important for the following reasons.

- It enables public health personnel to track the disease spread, vaccination, and adverse events across different sub-populations. It also allows researchers to gain new insights, and to scrutinize the data to understand the rationale behind the policies put forth by the government.
- Granular data is also more transparent and informative for the general public. Governments cannot give personalized health recommendations to each citizen. The government's advice on mask mandate, lockdowns, and vaccination is designed as a standard recommendation for all the citizens. However, health is a personal matter. If a person is more susceptible to COVID-19, then they need data on age, gender, comorbidities, AEFI (adverse events following immunization), etc., to make informed decisions.
- States in India are not isolated independent regions. People move from one state to another for work and leisure. Therefore, state governments should not just collect data and use them internally, but they should also publish the data so that anyone within the country can use them to make informed decisions.

## Methods

1. Additional information about methods and analysis would be helpful.

We have added additional text in the surveillance reporting section under Methods detailing the scoring process. Also, see our answers to questions 2 and 3 below.

Newly added text 1: (Lines 101-102 on Page 5)

These five categories indicate possible stages that a susceptible individual can go through during the pandemic.

Newly added text 2: (Lines 107-121 on Page 5)

During the assessment all indicators except the following two are scored either a 0 or a 1. The privacy indicator is scored a -1 if there is a privacy violation, else it is scored a 1. The “stratified by comorbidities indicator for deaths” is assigned a score of 1 if binary stratification (presence/ absence of comorbidity) of total deaths is reported. An additional score of 1 is given if more data such as stratification by a list of comorbidities or patient specific comorbidities are reported. We calculate two normalized scores for each subnational as described in [1]. One, a surveillance reporting score, which is the ratio of the total score earned by the subnational from all indicators and the maximum score possible from all indicators. Two, a granular surveillance reporting score, which is the ratio of the total score earned by the subnational from granular indicators and the maximum score possible from granular indicators. Both scores range between 0 (low) and 1 (high). During the calculation, the denominator is adjusted if any indicator does not apply to the subnational. For example, stratified by districts does not apply to Chandigarh because it does not have districts. Note that in [1], the surveillance reporting score is referred to as COVID-19 data reporting score and granular surveillance reporting score is referred to as granularity score.

2. Did one person code all of the websites or multiple people? If multiple people coded the websites, how did you ensure IRR?

At least two authors checked each platform independently on different days and arrived at a consensus on what data is being reported. We added lines 92-95 on Page 4 to clarify this.

Newly added text:

At least two authors checked each platform independently on different days and arrived at a consensus on what data is being reported. The complete list of digital platforms that we shortlisted and checked are made publicly available through the dataset released with this paper. See S1 Text for more details on data curation.

3. Surveillance Reporting – I’m not sure accessibility is the appropriate term or I would recommend better defining “accessibility”. Did you use Web Content Accessibility Guidelines or look at the reading levels of content presented?

The surveillance reporting indicators used in this study are from our prior work published in BMC Public Health.[[1]](https://www.zotero.org/google-docs/?bgeN2q) In the prior work, we proposed the indicators and grouped them into the four categories availability, accessibility, granularity and privacy. We defined / measured accessibility based on:

1) The ease of access to the webpage reporting data;
2) Availability of data in English; and
3) The presence of trend graphics.

We added lines 97-98 on Page 4 to make it more clear that the categories were defined in our prior work.[[1]](https://www.zotero.org/google-docs/?smZpLg)

Newly added text:

Vasudevan et al. developed a framework with 45 indicators to evaluate the reporting quality of COVID-19 surveillance data.[1] We use those indicators in the current study.

## Results

1. There are so many tables and figures (and figures within figures) that it is difficult to comprehend all the information. Consider removing or combining some tables/figures. Alternatively, consider turning this paper into multiple manuscripts.

Thank you for raising this concern. We have split the two figures into four. Each of the four figures showcases different aspects of data reporting and are self contained.

A. Fig 1 gives a high level summary of surveillance and vaccination reporting
B. Fig 2 focuses on granular surveillance, vaccination and bed availability data
C. Fig 3 highlights good data reporting practices from Nagaland
D. Fig 4 focuses on the magnitude of missing granular surveillance data and the differences in the reporting practices

2. Overall, additional text in the Results section would be helpful – rather than pointing to all the tables and figures.

Thank you for the suggestion. In addition to pointing to the tables and figures, we have added additional text in multiple places in the results section to improve readability. Also see our response to Q3 below

Newly added text 1: (Lines 176-177 in Page 7)

Nagaland also compares data from 2020 (first wave) with data from 2021 (second wave).

Newly added text 2: (Lines 180-185 in Page 7)

Subnationals that report both age and gender distribution for cases are: Nagaland, Odisha, Tamil Nadu, and Telangana. Similarly, subnationals that report both age and gender distribution for deaths are: Karnataka, Nagaland, Tamil Nadu, and Kerala. See Fig 4A–D for a compact summary of granular data availability and S3 Text for additional details. Even now, some subnationals do not report data stratified by districts. The tabular dataset released with this paper provides a comprehensive summary of what data each subnational is reporting.

Newly added text 3: (Lines 220-222 on Page 9)

They are Nagaland, Kerala, Odisha, Karnataka, Ladakh, Puducherry, Uttarakhand, Gujarat, Jharkhand, Madhya Pradesh, Mizoram, Punjab, Himachal Pradesh, and Manipur.

Newly added text 4: (Lines 227-231 on Page 9)

They are Haryana, Tamil Nadu, Kerala, Karnataka, Puducherry, Uttarakhand, West Bengal, Andhra Pradesh, Chhattisgarh, Gujarat, Madhya Pradesh, Punjab, Telangana, Rajasthan, Bihar, Chandigarh, Delhi, Goa, Himachal Pradesh, Dadra and Nagar Haveli and Daman and Diu. These results are also summarized in Fig 2.

3. Additionally, if the data are normally distributed, please report means and standard deviations or medians and ranges or interquartile ranges for data that are not normally distributed.

Data is not normally distributed. We have added a five number summary of the surveillance reporting score and the granular surveillance reporting score: minimum, first quartile, median, third quartile, and maximum.

Newly added text: (Lines 157-158 on Page 6)

The five number summary of the surveillance reporting score is, minimum = 0.33, first quartile = 0.39, median = 0.46, third quartile = 0.49, and maximum = 0.61.

Newly added text: (Lines 172-173 on Page 7)

The five number summary of the surveillance reporting score is, minimum = 0, first quartile = 0, median = 0.17, third quartile = 0.22, and maximum = 0.50.

## Discussion

The “so what” and implications of this study need to be strengthened. The commentary on the results reads awkwardly and is a bit of a stretch in the current state.

We have strengthened the implications of this study by adding additional text to the discussion section. Also see lines 56-68 on Page 3 in Introduction.

Newly added text 1: (Lines 252-260 on Page 10)

This limitation has important implications.

1. It is difficult to understand the effect of the virus on the age group below 18 without data on the age distribution of cases, deaths, and ICU cases. This is important as schools are reopening in India.
2. Number of new confirmed cases per 100,000 population per week and number of COVID-19-attributed deaths per 100,000 population per week are two primary indicators to assess the level of community transmission as per WHO. It is important to track these indicators at the district level because subnationals in India are big and health care facilities vary significantly within a subnational. Therefore, states should publish data at least at the district level.

Newly added text 2: (Lines 284-286 on Page 10)

For example, it might be possible to infer religion from names of some people and target specific groups leading to communal violence. India has already seen communal violence in the context of COVID-19.

# References

[1.	Vasudevan V, Gnanasekaran A, Sankar V, Vasudevan SA, Zou J. Disparity in the quality of COVID-19 data reporting across India. BMC Public Health. 2021 Jun 24;21(1):1211.](https://www.zotero.org/google-docs/?PLHoiq)
