# Supplementary material for: Assessment of COVID-19 data reporting in 100+ websites and apps in India
Source: PLOS Glob Public Health. 2022 Apr 15;2(4):e0000329. doi: 10.1371/journal.pgph.0000329 (PMC10022220; doi:10.1371/journal.pgph.0000329)
Supplement: Attachment — Submitted filename: Response to Reviewers.docx [file pgph.0000329.s003.docx]

Response to Reviews

**Title**: Assessment of COVID-19 data reporting in 100+ websites and apps in India

**Authors**: Varun Vasudevan, Abeynaya Gnanasekaran, Bhavik Bansal, Chandrakant Lahariya, Giridara Gopal Parameswaran, James Zou

**Date:** Dec 28, 2021

# Journal Requirements

Please review your reference list to ensure that it is complete and correct. If you have cited papers that have been retracted, please include the rationale for doing so in the manuscript text, or remove these references and replace them with relevant current references. Any changes to the reference list should be mentioned in the rebuttal letter that accompanies your revised manuscript. If you need to cite a retracted article, indicate the article’s retracted status in the References list and also include a citation and full reference for the retraction notice.

We checked our reference list for completeness and correctness. We added a new reference (Ref 22) in the discussion section to address a question from Reviewer 4.

# Additional Editor Comments

Please address all reviewer inputs, particularly the minor suggestions from reviewer 4 in your final version.

We have addressed all the suggestions from both reviewers. Please see below.

# Reviewer 3

1. This manuscript does a very good job of objectively assessing the quality and granularity of COVID-19 related data released by states and union territories of India. It reads well as a continuation of the previous article in BMC Public Health in which the COVID-19 Data Reporting Score was developed. In addition to focusing on granularity of surveillance data, the present manuscript also analyzes vaccine related data and bed vacancy data. "Lack of data" is a widely heard complaint and the figures included in this manuscript illustrate what that really means. While systematically and painstakingly scoring and analyzing each subnation on one side, the authors have also highlighted the best and the worst practices with specific examples. The methodology is simple and straightforward although it requires a couple of careful readings of the textual description to realize that. It is possibly an unfortunate constraint of the medium of publication that useful details are spread across multiple locations (text, figure, supplementary file, and data repository).

Thank you for the positive comments and feedback which have been helpful in improving this manuscript. Please refer to our point-by-point responses below.

2. The "men and women" in the opening sentence seems to reinforce an outdated binary.

Thank you for the comment. We have modified the opening sentence as follows.

**Modified sentence:** (See Page 2, line 45)

How many people of each gender have died due to COVID-19 in India?

3. The color palette in Fig 1A is not very friendly to this reviewer who has red-green color deficiency.

Thank you for pointing it out. We have changed the palette to a colorblind friendly orange-blue diverging palette.

4. It is not stated whether the authors have pointed out the privacy violations to the concerned authorities and given them a chance to rectify.

We have reached out to two national organizations in India. We had private communications over email with the members of NITI Aayog (National Institution for Transforming India) and NIDM (National Institute of Disaster Management) during the period August–September 2021. These organizations have not concretely responded to our concerns yet; we hope the publication of this manuscript will further highlight the issues.

5. This manuscript legitimizes in academic terms the rather political demand for open data. And the poise with which the authors have done so makes it an important contribution in the field of open data for health governance and public policy.

Thank you for the feedback.

# Reviewer 4

## Overall Comments

1. The authors have used a novel and reproducible approach to demonstrate how the COVID-19 data is reported. The topic is relevant to the current context and informs readers about how the data is being used. Since the authors have accessed various websites for shortlisting digital platforms, it would be required for them to mention the exact time and date that these websites were accessed, as websites evolve over a period of time (dynamic in nature).

Thank you very much for your helpful feedback. All the websites and mobile apps were accessed over the 15 day period from 22 May to 5 June 2021. We have stated the dates in the methods section. During this period, we accessed the platforms multiple times both from India and USA. At least two authors checked each platform independently on different days and arrived at a consensus on what data is being reported. We followed this procedure to ensure that a non-zero score was assigned to an indicator only when the state was consistent in their reporting during this period.

## Methods

1. Written in a detailed and reproducible manner.

Thank you for the feedback.

2. Line 81: It would be good to provide a definition for web-based and mobile based digital platforms along with a few examples as most platforms these days are web-based, but accessible through mobile. I am sharing a link that may be referred to <https://careerfoundry.com/en/blog/web-development/what-is-the-difference-between-a-mobile-app-and-a-web-app/>

Thank you for the feedback. Our usage of the word mobile apps is consistent with the definition in the link you provided. To clarify this, we have added the following sentence in the Methods section.

**Newly added text:** (See Page 4, lines 88-89)

Here, digital platforms refer to websites and mobile applications designed for Android / iOS.

## Discussions

1. In the methods section the authors mention that Vasudevan et al., developed a framework of 45 indicators to evaluate the reporting quality of COVID-19 surveillance data. Did the authors explore if the framework has been used for settings other than India? The authors may bring the findings of those studies (if any) in the discussion section. It would be imperative to cite studies from other regions on this topic (if any). If there are not many studies, then point towards the paucity in literature and that adds value to your research.

Thank you for the great question. No, the exact framework has not been used in any other country for evaluating the reporting quality of COVID-19 surveillance data. A recent publication from Rocco et. al,^[1]^ (published on Dec 1, 2021) uses 13 indicators to evaluate the quality of COVID-19 surveillance data in 15 federal democracies. This is one of the few works that study the quality of data reporting. We added a new paragraph in the discussion section with a few sentences on this study.

**Newly added paragraph:** (See Page 12, lines 313-319)

A similar study performed by Rocco et al. evaluated the quality of COVID-19 surveillance data across 15 federal democracies, including India.[22] Their evaluation using 13 indicators found a statistically significant association between subnational data quality and critical public health system capacity indicators. Countries such as the United States, Canada, Belgium, and Germany that are known to have substantial public health capacity and infrastructure scored higher on data quality. In contrast, countries like Argentina, India, and Malaysia scored significantly below the median score.

## References:

1. The reference 6 and 18 need attention. "The Lancet" should not be written as "Lancet T".

Thank you for pointing it out. We have corrected both reference 6 and 18.

# Additional Comment

While revising your submission, please upload your figure files to the Preflight Analysis and Conversion Engine (PACE) digital diagnostic tool, https://pacev2.apexcovantage.com/. PACE helps ensure that figures meet PLOS requirements. To use PACE, you must first register as a user. Registration is free. Then, login and navigate to the UPLOAD tab, where you will find detailed instructions on how to use the tool. If you encounter any issues or have any questions when using PACE, please email PLOS at figures@plos.org. Please note that Supporting Information files do not need this step.

Thank you. We have used PACE to convert our figures and meet PLOS requirements.

1. Rocco P, Rich JA, Klasa K, Dubin KA, Béland D. Who Counts Where? COVID-19 Surveillance in Federal Countries. Journal of Health Politics, Policy and Law. 2021 May 21.
